# Solution Ionic Strength Can Modulate Functional Loop Conformations in *E. coli* Dihydrofolate Reductase

**DOI:** 10.1021/acs.jpcb.4c00677

**Published:** 2024-04-23

**Authors:** C. Satheesan Babu, Jih-Ying Chen, Carmay Lim

**Affiliations:** Institute of Biomedical Sciences, Academia Sinica, Taipei 11529, Taiwan

## Abstract

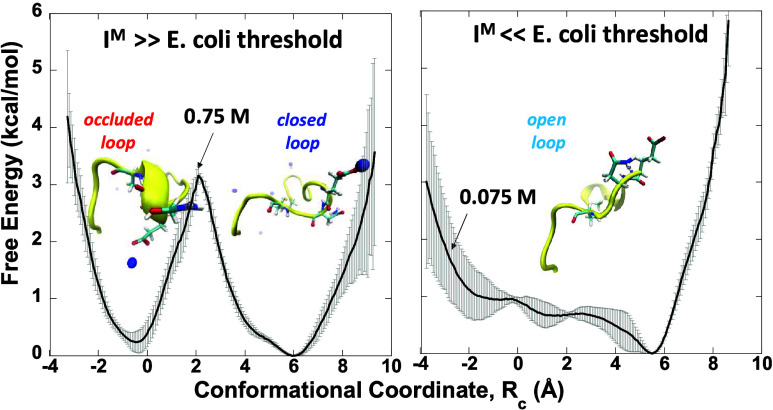

The observation of multiple conformations of a functional loop (termed M20) in the *Escherichia coli* dihydrofolate reductase (*ec*DHFR) enzyme triggered the proposition that large-scale motions of protein structural elements contribute to enzyme catalysis. The transition of the M20 loop from a *closed* conformation to an *occluded* conformation was thought to aid the rate-limiting release of the products. However, the influence of charged species in the solution environment on the observed M20 loop conformations, independent of charged ligands bound to the enzyme, had not been considered. Molecular dynamics simulations of *ec*DHFR in model CaCl_2_ solutions of varying molar ionic strengths *I*^M^ reveal a substantial free energy barrier between *occluded* and *closed* M20 loop states at *I*^M^ exceeding the *E. coli* threshold (∼0.24 M). This barrier may facilitate crystallization of *ec*DHFR in the *occluded* state, consistent with *ec*DHFR structures obtained at *I*^M^ exceeding 0.3 M. At lower *I*^M^ (≤0.15 M), the M20 loop can explore the *occluded* state, but prefers an *open*/*partially closed* conformation, again consistent with *ec*DHFR structures. Our findings caution against using *ec*DHFR structures obtained at nonphysiological ionic strengths in interpreting catalytic events or in structure-based drug design.

## Introduction

Enzymes exhibit a remarkable ability to accelerate the rate of chemical reactions that would otherwise proceed slowly in solution. They achieve this by serving (i) a *structural* role, stabilizing the rate-limiting transition state of a reaction,^1−5^ or destabilizing the ground state of the reactants^6−11^ and/or (ii) a *dynamical* role, increasing the transmission coefficient.^12−15^ Another dynamical role that has been proposed is the large-scale motions of secondary structural elements, occurring on time scales matching the enzyme’s catalytic rate, that are thought to optimize enzyme proficiency by facilitating reactant and product binding/unbinding. The enzyme, *Escherichia coli* dihydrofolate reductase (*ec*DHFR), has served as a paradigm for such large-scale dynamical effects:^16−18^ Structural studies, including X-ray,^19,20^ NMR,^17,21−24^ and neutron diffraction,^25^ show the M20-containing loop (residues 9–24) in a *closed* conformation when bound to reactant analogues, but in an *occluded* conformation when bound to products.^19^ Consequently, large-scale motion of the M20 loop has been proposed to facilitate reactant and product binding/unbinding during the enzyme-catalyzed reaction.^17^ This study addresses an overlooked factor: the sensitivity of functional loop conformations to multivalent ions in a buffer solution. These ions of charge *Z_i_* and concentration *c_i_* contribute to the molar ionic strength (*I*^M^) according to
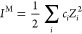
1

Importantly, this study highlights the fact that catalytic loop conformations in structures solved at an *I*^M^ beyond an organism’s tolerance may not represent *in vivo* catalytic events.

**Scheme 1:**
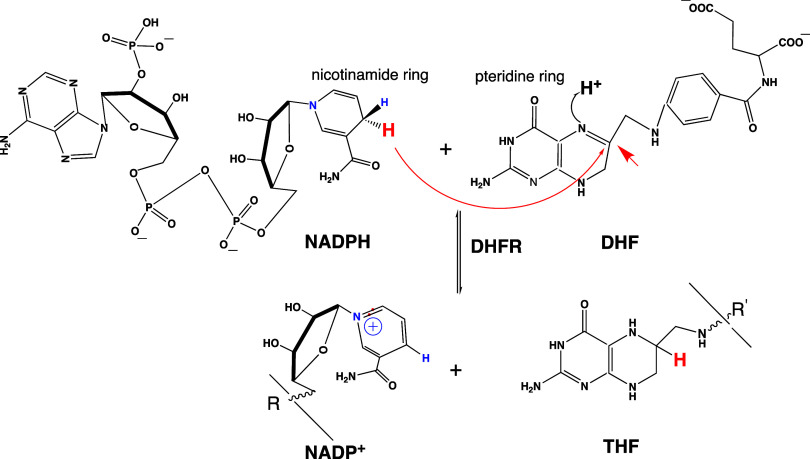
DHFR Reaction Scheme Hydride transfer from the NADPH coenzyme to dihydrofolate (DHF) substrate yielding NADP^+^ and tetrahydrofolate (THF) products catalyzed by the DHFR enzyme. The curved arrow shows the transfer of H^–^ (red) from the nicotinamide ring of NADPH to the pteridine ring of DHF.

DHFR, a key enzyme in the folate cycle needed for DNA synthesis,^26^ is a target for approved anticancer and antibiotics/antiparasitic drugs including methotrexate, trimethoprim, and pyrimethamine.^25,27−31^ DHFR catalyzes the reduction of dihydrofolate (DHF) to tetrahydrofolate (THF) with the assistance of nicotinamide adenine dinucleotide phosphate (NADPH), which serves as a hydride (H^–^) donor and is oxidized to NADP^+^ during the reaction (Scheme 1). This chemical transformation involves not only the binding and unbinding of large, flexible NADPH and NADP^+^, which prefer different conformations in solution^32^ but also the rate-limiting release of the product THF. The search for factors that could assist DHFR to efficiently bind NADPH and DHF reactants as well as unbind NADP^+^ and THF products led to a catalytic role of the M20 loop based on distinct conformations seen in *ec*DHFR structures.

**Figure 1:**
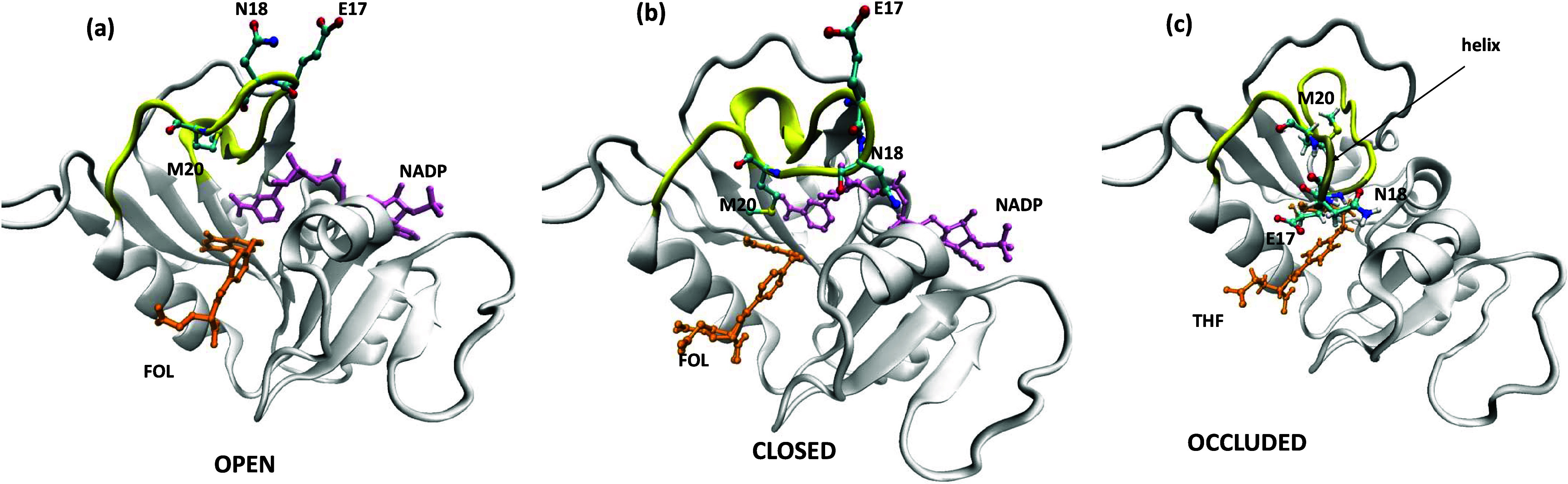
Representative X-ray structures of *ec*DHFR with the M20 loop in three distinct ordered conformations: (a) *open* (PDB 1ra2), (b) *closed* (PDB 3ql3), and (c) *occluded* (PDB 6cw7). The M20 loop (residues 9–24) is in yellow with the residues comprising the turn (E17, N18, and M20) in ball and stick, NADP is in pink, and the substrate analogue (folate) as well as the product THF are in orange.

The numerous *ec*DHFR structures, loaded with substrates, inhibitors, or coenzymes as binary or model Michaelis complexes, in the Protein Data Bank (PDB)^33^ show that the M20 loop can adopt multiple conformations including *open* (*partially closed*), *closed*, or *occluded*, as illustrated by representative PDB structures in Figure 1a–c. With NADP^+^ and folate bound in the PDB 1ra2(19) structure (Figure 1a), an *open* M20 loop (yellow) conformation is seen with the E17, N18, and M20 side chains pointing away from the coenzyme/substrate-binding site, creating space for solvent molecules and ligands to enter the active site. With the same NADP^+^ and folate ligands bound in the PDB 3ql3(17) structure (Figure 1b), a *closed* M20 loop conformation is seen with the N18 side chain oriented toward the coenzyme-binding site. On the other hand, with only the product THF bound in the PDB 6cw7(20) structure (Figure 1c), an *occluded* conformation is seen with the E17 side chain oriented toward the substrate-binding site, in contrast to the *open* or *closed* conformer.

**Scheme 2:**
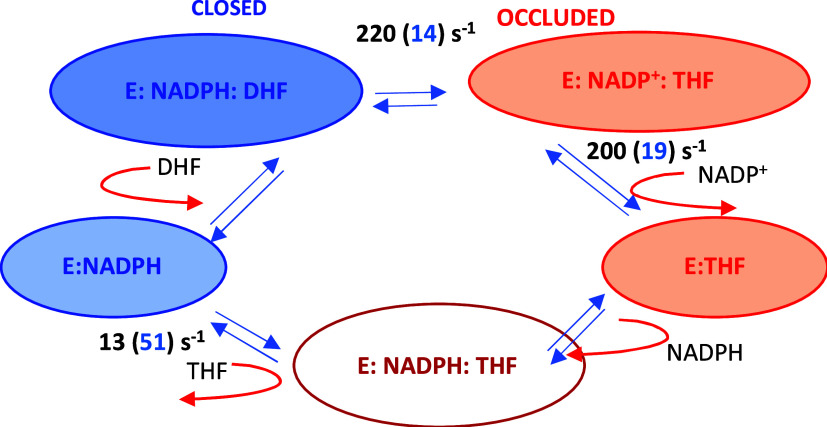
Proposed Catalytic Cycle Involving the Transition of *Closed* to *Occluded* M20 Loop upon Reaction^17^^,^ The numbers over the arrows are forward rates of wild-type *ec*DHFR and those in parentheses are those for a dynamic-knock-out N23PP/S148A mutant enzyme (see text).

The predominance of *closed* M20 loop conformations in reactant-bound *ec*DHFR models and *occluded* conformations in product-bound models^17,19,20^ led to the proposal that a *closed*-to-*occluded* transition accompanied the chemical step to promote NADP^+^ coenzyme release by occluding the nicotinamide-binding site (Scheme 2). Subsequent NADPH binding to an *occluded* loop is thought to result in a steric clash with the pterin ring, facilitating the release of product THF. This reverts the M20 loop to the *closed* state, enabling binding of another DHF substrate, completing the catalytic cycle.^34^ The proposed dynamical role of the M20 loop in the catalytic cycle was supported by the finding that a knockout mutant N23PP/S148A, which hampers the M20 loop conformational flexibility, drastically reduced the rates of the chemical reaction and the NADP^+^ release but increased the rate for product release (see numbers in parentheses in Scheme 2).^17^ Another mutant M42W/G121V involving residues in van der Waals contact with the M20 loop produced similar effects.^35^ Therefore, the motion of the M20 loop has been touted as an example of large-scale dynamic effects in enzyme catalysis.^17^

An assumption underlying the proposed role of large-scale dynamical effects in catalysis is that the M20 loop conformations seen in the experimental structures depend only on the type of ligand bound to *ec*DHFR. The possibility that the M20 loop conformations may be influenced by external factors such as charged species in the solution environment had not been considered. However, computational studies found that the M20 loop stability is sensitive to the sudden change of electrostatic environment during the catalyzed bond formation and breaking process.^35^ Our previous study showed that the M20 loop conformations are sensitive to the treatment of charge–charge interactions in molecular dynamics (MD) simulations.^36^ Furthermore, we noted that the M20 loop is *open* in crystal structures obtained at low ionic strength but *closed* or *occluded* in structures solved using relatively high ionic strength.^37^ For example, the *ec*DHFR structure obtained using 10 mM CaCl_2_ (*I*^M^ = 0.03M) shows an *open* M20 loop, but that solved using 100 mM CaCl_2_ (*I*^M^ = 0.3 M) shows a *closed* loop.^38^ In solution-phase NMR experiments, the ionic strength can be raised by potassium phosphates or citrates in the buffer solution.^21^ However, the intracellular *I*^M^ cells in *E. coli* has an upper limit (∼0.24 M),^39^ so loop conformations seen in structures solved at *I*^M^ exceeding this limit may lack functional relevance in catalysis.^40^

We hypothesized that the ionic strength of the solution environment may influence the observed M20 loop conformations. To isolate the effects of solution ionic strength, we focused on the apoenzyme, as the presence of ligands bound to the enzyme makes it difficult to distinguish between the effects of charged bound ligands and charged ions in the solution medium on the M20 loop conformations. We performed long MD simulations starting from the well-defined *ec*DHFR X-ray structures of the M20 loop in the *closed*, *open*, and *occluded* states for various ionic strengths, along with biased (umbrella) sampling simulations in regions of importance. Based on the 3 sets of simulations at each ionic strength, we computed the free energy barrier separating the distinct loop conformations as a function of a conformational coordinate *R*_c_ that could describe the transitions between *closed*, *open*, and *occluded* M20 loop conformations.^37^ Our results show that a relatively high ionic strength creates a significant free energy barrier, stabilizing the M20 loop in the *occluded* or *closed* state. This stabilization facilitates crystallization in these two states. Our findings emphasize the importance of considering ionic strength effects on functional loop conformations in enzyme catalysis and drug design.

## Methods

### Definition of the Reaction Plane

**Figure 2:**
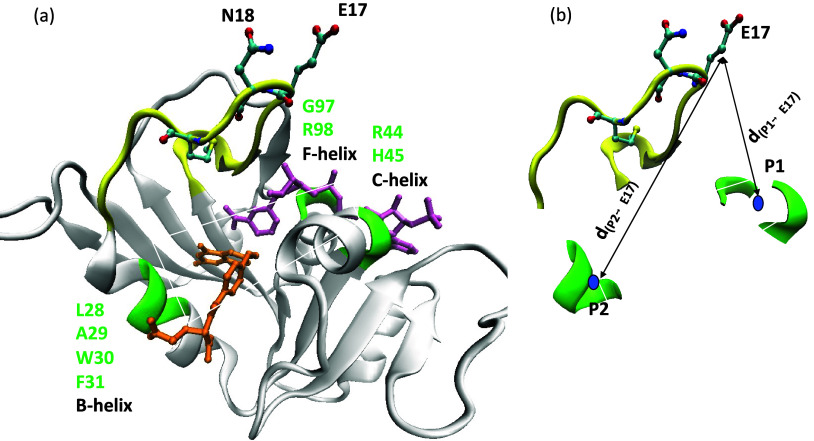
Conformational coordinate, *R*_c_, defining transitions among *open*, *closed*, and *occluded* conformers. (a) The reaction plane (shaded) links residues (green) on substrate-binding helix B and coenzyme-binding helices C and F in the PDB entry 1ra2 structure with an *open* M20 loop (yellow). The E17 and N18 side chains are in ball and stick. (b) The arrows indicate distances from *P*1 and *P*2 to the geometric center of the side chain heavy atoms of E17.

As shown in Figure 2a, the coenzyme NADPH (pink sticks) binds at the Rossmann-fold cleft^41^ with its pyrophosphate sandwiched between helices C and F. The substrate analogue (folate, orange sticks) is bound with its pteridine ring optimally oriented for hydride transfer to the nicotinamide ring of NADPH through the space between helices B and C. We defined a reaction plane where the NADPH nicotinamide ring is optimally oriented to transfer a hydride to the substrate’s pteridine ring by the geometric average of the C^α^ atoms of (i) ^28^LAWF^31^ in the B-helix, (ii) R44 and H45 in the C-helix, and (iii) G97 and R98 in the F-helix.

### Conformational Coordinate, *R*_c_

In our previous work,^37^ we introduced a conformational coordinate *R*_c_ that takes into account the M20 loop orientation relative to key amino acid residues on the cofactor-binding helices C and F and the substrate-binding helix B. It is defined by eq 2,
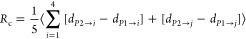
2where point *P*1 represents the cofactor-binding site, *P*2 represents the substrate-binding site, *j* indicates the point where the loop turns around, and *i* corresponds to loop residues E17, N18, M20, or P21. *P*1 is the geometric average of the C^α^ atoms from residues R44, H45, G97, and R98, located on the Rossmann-fold helices C and F; *P*2 is the geometric average of residues ^28^LAWF^31^ on the B-helix; point *j* is the geometric average of E17 and N18 C^α^ atoms; point *i* is the geometric center of the side chain heavy atoms comprising loop residue *i* (E17, N18, M20, or P21) and *d*_*P*1/*P*2→*j*_/*d*_*P*1/*P*2→*i*_ denotes the distance from *P*1/*P*2 to *j* or *i*, (Figure 2b).

### Molecular Dynamics (MD) Simulations

All simulations and free energy calculations were conducted at 298.16 K and 1 atm pressure using the CHARMM42 program^42^ with a time step of 2 fs. The CHARMM36 all-atom force field^43^ was used for modeling protein atoms and ions, and the TIP3P model^44^ for water molecules. Simulations were performed in a 93 × 93 × 93 Å^3^ periodic box,^36,37^ and long-range electrostatic forces were treated using the particle mesh Ewald method^45^ with nonbonded potentials truncated at 12 Å.

### Simulations of Solutions at Different Ionic Strengths

Since most *ec*DHFR PDB structures were solved using CaCl_2_ as a buffer component, we prepared model CaCl_2_ solutions in TIP3P water of *I*^M^ spanning the range typically used in crystallization buffers. Using eq 1, the *I*^M^ values for CaCl_2_ concentrations of 0.025, 0.05, 0.10, and 0.25, M were 0.075, 0.15, 0.30, and 0.75 M, respectively. The ionic solutions employed a 93 × 93 × 93 Å^3^ cubic box containing well-equilibrated TIP3P^44^ water molecules. One Ca^2+^ and two Cl^–^ ions were randomly distributed to circumvent the artificial clustering of ions at the start of the simulations. A new ion was then placed outside a 15 Å radius sphere of the present ion, avoiding overlap with any TIP3P molecules. Each ionic solution system was energy-minimized in cycles of 5000 steps of steepest descent followed by 5000 steps of Newton–Raphson. It was then subjected to 6 ns of equilibration and 12 ns of production dynamics. The resulting solvation boxes of different ionic strengths were used in simulations of the enzyme–ion–water systems below.

### Simulations of *ec*DHFR at Different Ionic Strengths

The simulations started from three well-characterized X-ray structures of *ec*DHFR with the M20 loop in the *closed* (3ql3),^17^*open* (1ra2),^19^ or *occluded* (6cw7)^20^ conformation. Protonation states of ionizable residues were assigned using available experimental p*K*_a_ values.^46^ Thus, H124 was protonated, whereas other His residues (H45, H114, H149) were assumed to be neutral with only the N^ε2^ protonated based on their ring orientations. The HBUILD module in the CHARMM42 program^42^ was used to build any missing H atoms in the crystal structure. Each structure was centered in one of the equilibrated ionic solution boxes (see above). TIP3P molecules that fell inside a sphere of 2.8 Å around a protein-heavy atom were removed. This yielded a solvated protein system consisting of 2493 protein atoms and 26,060 TIP3P water molecules. Ions that overlapped with the protein region were redistributed to bulk solution by applying the above ion distribution algorithm. The total number of ions was adjusted to maintain a neutral solvated protein system and the numbers of Ca^2+^ and Cl^–^ ions were used to compute the ionic strength according to eq 1. Each neutral solvated protein system at a given ionic strength underwent three simulations starting from the *closed* (3ql3), *open* (1ra2), and *occluded* (6cw7) X-ray structures, yielding a total of 12 simulations across four different ionic strengths.

For each simulation, the solvent and ions were energy-minimized with the protein solute fixed using the adopted basis Newton–Raphson method for ∼2000 steps, followed by one ns of equilibration. Subsequently, only the protein backbone atoms were fixed, while the solvated protein side chains were energy-minimized and equilibrated for one ns. Finally, the entire system (protein, water, and ions) was energy-minimized and equilibrated for 6 ns, followed by 70 ns of production dynamics. Each production trajectory was stored for analysis.

### Computation of Free Energy Profiles

The *open* state has been identified as a high-energy conformer^47^ compared to the *closed* and *occluded* forms. Since the *open* conformer is well-defined in the PDB 1ra2 structure,^19^ our strategy in computing the free energy profiles involved conducting three independent simulations, each of length 100 ns, starting from the *ec*DHFR crystal structures of the *occluded* (PDB 6cw7),^20^*closed* (PDB 3ql3),^17^ and *open* (PDB 1ra2)^19^ M20 loop. The free energy or the potential of mean force at a specific conformation coordinate *R*_c_ was computed from
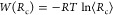
3where *R* is the molar gas constant, *T* is the temperature, and ρ⟨*R*_c_⟩ is the relative probability density that the system is found in the interval between *R*_c_ and δ*R*_c_. As the *open*, *closed*, and *occluded* simulation systems are identical with the same numbers of protein atoms, TIP3P water molecules, and ions, we counted the number of configurations within a given *R*_c_ range, i.e., ρ⟨*R*_c_⟩, from all of the three simulations. Knowing ρ⟨*R*_c_⟩, the free energy profile, *W*(*R*_c_), was constructed using eq 3. The weighted histogram analysis method (WHAM) program^48^ was employed to join the *R*_c_ populations to yield a single free energy curve spanning all three M20 loop states. Free energy profiles, *W*(*R*_c_), were obtained for all systems at *I*^M^ = 0.075, 0.15, 0.30, 0.75, 1.50, and 3 M. However, at an *I*^M^ of 0.75 M, the free energy barrier to reach the *occluded* state from the *closed* state increased to ∼3.5 kcal/mol, suggesting potential sampling problems in this region. To verify the accuracy of the free energies in this region, umbrella sampling free energy simulations were performed, introducing umbrella potentials of 10 kcal/mol/Å at intervals of 0.2 Å near the barrier region. The *W*(*R*_c_) was then generated using the WHAM program.^48^ Comparison between biased and unbiased free energy profiles at an *I*^M^ of 0.75 M showed agreement (Supporting Information, Figure S1), indicating that our unbiased approach yields the correct free energy profiles.

### Computation of Electrostatic Potentials

We computed electrostatic potentials at a reference point on the loop, which is the geometric center of the C^α^ atoms of residues E17, N18, and M20. The distance-dependent electrostatic potential due to ions, Φ^α^(*R*)^ion^, is given by^49^
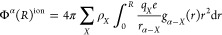
4

In eq 4, ρ_*X*_ is the density of ion *X* (Ca^2+^ or Cl^–^), *g*_∝–*X*_(*r*) is the radial pair distribution function of ion *X* from point α at distance *r*, *q*_*X*_ is the charge of particle *X*, *e* is the electronic charge, and *R* is the radius of the sphere. These computations used particle summations, as discussed in our earlier work.^50^ In analogy to Φ^α^(*R*)^ion^, we also computed the contributions from solvent atoms, Φ^α^(*R*)^solv^ to the total potential, Φ^α^(*R*).

## Results

### Distinguishing M20 Loop Conformations by the Reaction Plane

**Figure 3:**
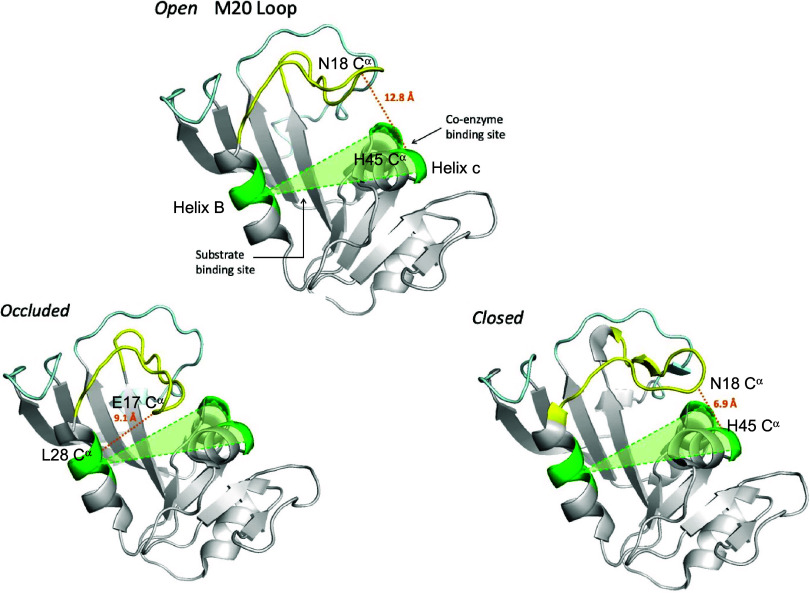
Three distinct M20 loop conformations in relation to the reaction plane. Representative *open* structure (PDB 1ra2),^19^*closed* structure (PDB 3ql3),^17^ and *occluded* structure (PDB 6cw7)^20^ with the reaction plane in green. The *occluded* conformer differs from the *open* or *closed* conformer by the proximity of E17 to the substrate-binding site, whereas the *closed* state differs from the *open* conformer by the proximity of N18 to the coenzyme-binding site.

M20 loop conformations have been classified via hierarchical clustering of 162 human and *E. coli* DHFR PDB structures using the root-mean-square deviation of the M20 loop backbone C^α^ atoms.^20^ Here, we present a way to visually distinguish these different M20 loop conformations based on the distance/orientation of the M20 loop “nose” comprising E17 and N18 relative to a plane encompassing the substrate- and coenzyme-binding site. To identify defining features of the 3 M20 loop conformations, we pairwise aligned all *ec*DHFR PDB structures onto the representative *closed*, *open*, and *occluded* structures in Figure 1 using PyMol.^51^ A distinguishing characteristic of the *occluded* conformer is the orientation of the M20 loop nose toward the substrate-binding site; hence E17 is closer to L28 on helix B than observed in the *open* or *closed* conformer (Figure 3). In contrast, the M20 loop nose is positioned above the coenzyme-binding site in the *open* and *closed* states, but it is closer to the coenzyme-binding site in the *closed* conformer. Consequently, N18 is much further from H45 on helix C (∼11–13 Å) in the *open* state than in the *closed* loop (∼7 Å).^36^

### Correlation between M20 Loop Conformations and the Solution Ionic Strength

**Figure 4:**
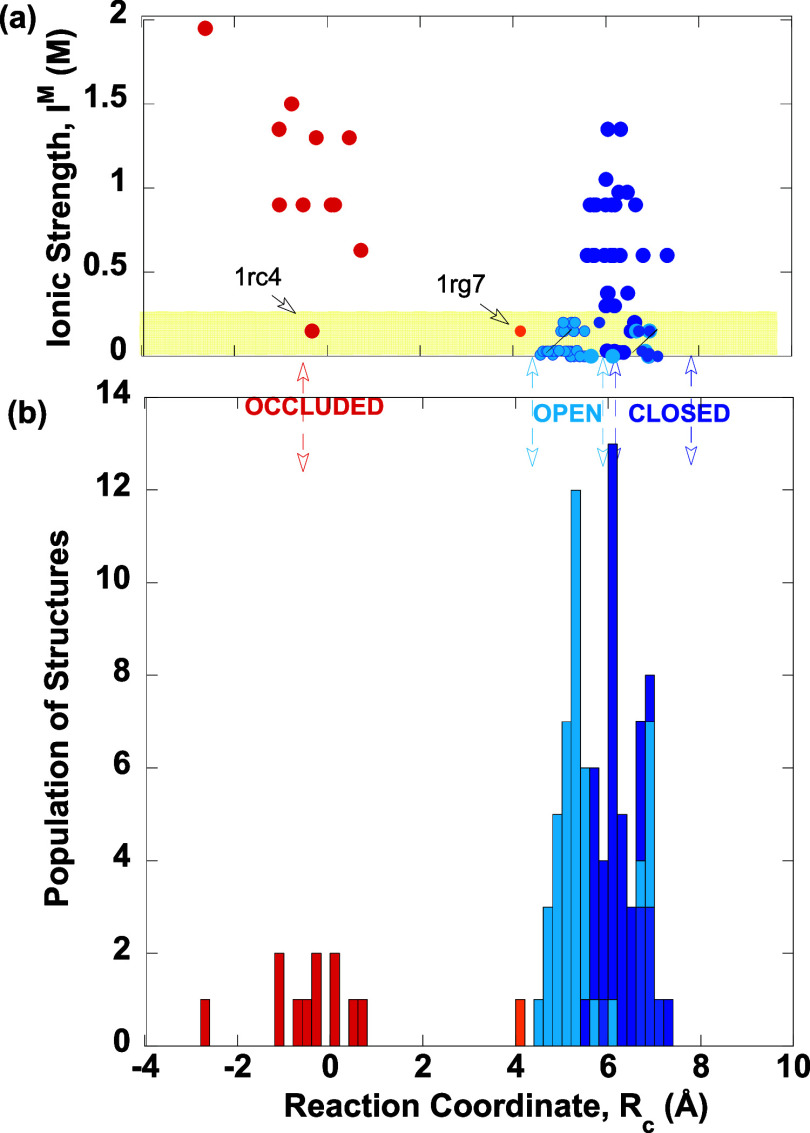
Correlation between the crystallization buffer *I*^M^ and the observed M20 loop conformations in ecDHFR structures. (a) *Occluded* (red), *open* (cyan), and *closed* (blue) M20 loop conformations classified using hierarchical clustering^20^ in *ec*DHFR PDB structures solved at ambient temperatures and pressures and various ionic strengths *I*^M^ as a function of *R*_c_. The PDB entry 1rg7, which has an M20 loop conformation in between *occluded* and *closed*, is in orange. The yellow-shaded region corresponds to *I*^M^ < 0.25 M. (b) Number of *ec*DHFR PDB structures as a function of *R*_c_.

To assess the influence of *I*^M^ on the M20 loop conformation, we extracted the 86 *ec*DHFR structures used in hierarchical clustering by Cao et al.,^20^ excluding structures obtained at extreme conditions (low temperature or high pressure) that do not reflect cellular conditions. The PDB entries and corresponding references provide information on the type and concentration of multivalent salts used as buffer components during crystallization. Structures solved using only 2:1 salt were included, as the simulations were performed in CaCl_2_ solutions. Supporting Information Table S1a–c list the PDB entries, resolution, pH, type and concentration of salt, the *I*^M^ computed using eq 1, and the corresponding *R*_c_ values computed using eq 2 for the *occluded*, *open*, and *closed* M20 loop conformations classified by Cao et al.^20^Figure 4a,b shows the crystallization ionic strength and number of the *ec*DHFR structures against the *R*_c_, where the *occluded*, *open*, and *closed* M20 loop conformations are in red, cyan, and blue, respectively. Note that although the distance between N18 and H45 C^α^ atoms can differentiate *open* and *closed* conformers (Figure 3),^37^ it cannot distinguish *occluded* and *closed* conformers. However, the current choice of *R*_c_ can separate *occluded* conformers from *open* and *closed* ones.

The results in Figure 4 show an apparent correlation between the crystallization buffer *I*^M^ and the observed M20 loop conformations in the *ec*DHFR structures. Most M20 loop conformers were crystallized in buffers containing salt solutions with an *I*^M^ exceeding the *E. coli* intracellular threshold of 0.24 M.^39^ Notably, all *occluded* M20 loop structures were obtained at *I*^M^ ≥ 0.5 M, except for PDB entries 1rc4 and 1rg7, which were obtained using CaCl_2_ with an *I*^M^ of 0.15 M.^19^ These *occluded* structures typically exhibit *R*_c_ < 2 Å except PDB entry 1rg7, which has a *R*_c_ of 4.1 Å. In the 1rg7 structure, the side chains of E17 to M20 are disordered; however, the E17 backbone amide points toward the coenzyme-binding site rather than the substrate-binding site, resulting in a much longer C^α^(E17)–C^α^(L28) distance than typically seen in *occluded* structures.

In contrast, *open* M20 loop structures, with *R*_c_ ranging from 4.6 to 5.7 Å, were obtained at *I*^M^ below the *E. coli* threshold. Dimeric *ec*DHFR structures, displaying both *closed* and *open* M20 loop conformations, were also obtained at *I*^M^ < 0.25 M, but the *open* conformer within the dimer had *R*_c_ > 5.6 Å. *Closed* M20 loop structures, with *R*_c_ ranging from 5.8 to 7.4 Å, were generally crystallized under high *I*^M^, typically in the 0.25–1.5 M range.

In summary, our analysis of PDB structures suggests that *I*^M^ can influence the stability of the observed M20 loop conformations. High ionic strength (*I*^M^ > 0.25 M) in the crystallization buffer predominantly yielded *ec*DHFR X-ray structures exhibiting *closed* or *occluded* M20 loops, suggesting that a high concentration of 2:1 salt, such as CaCl_2_, in the crystallization buffer may contribute to stabilizing these conformations. In contrast, at physiological ionic strengths (*I*^M^ < 0.25 M), *ec*DHFR X-ray structures exhibited mostly *open* or *closed* M20 loops.

### Conformational Free Energies

To determine if and how the *I*^M^ affects the stability of the M20 loop conformations, we computed potentials of mean forces, *W*(*R*_c_), from simulations of *ec*DHFR in TIP3P water at different ionic strengths (*I*^M^ = 0.075, 0.15, 0.30, and 0.75 M), taking into account protein–protein, protein–water, protein–ion, water–water, water–ion, and ion–ion interactions, as described in the Methods section. Below, we present the free energy profiles for physiological (*I*^M^ < 0.25 M) and nonphysiological (*I*^M^ > 0.25 M) ionic strengths. We correlate the free energy profiles with the *open* and *closed* M20 loop conformations classified by Cao et al.^20^ using hierarchical clustering, taken from Figure 4a, for the respective *I*^M^.

#### *I*^M^ = 0.075 M

**Figure 5:**
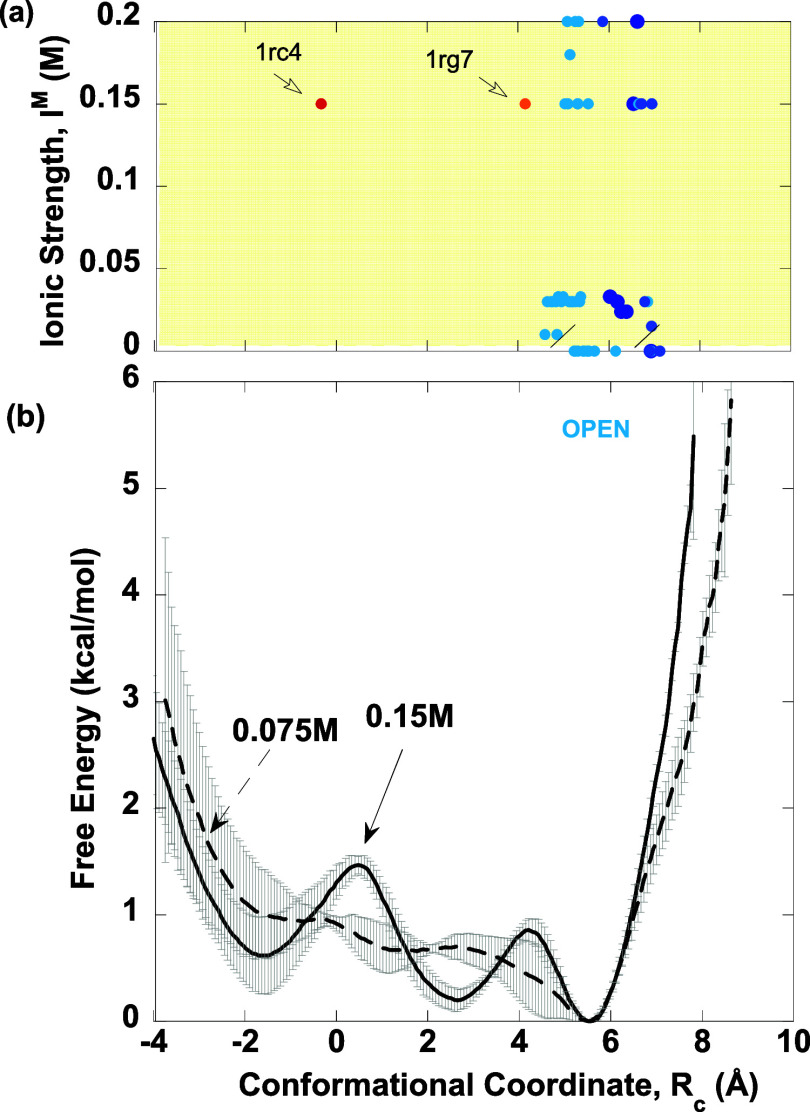
Correlation of free energy profiles for low *I*^M^ (0.075 and 0.15 M) with the *ec*DHFR structures solved at physiological *I*^M^. (a) *Open* (cyan) and *closed* (blue) M20 loop conformations classified using hierarchical clustering^20^ in *ec*DHFR PDB structures solved at *I*^M^ < 0.25 M from Figure 4a. (b) Free energy as a function of *R*_c_ for *I*^M^ = 0.075 ^M^ (dashed curve) and 0.15 M (solid curve) in CaCl_2_ solution. The gray bars represent the error bars.

At the lowest *I*^M^ studied of 0.075 M, the free energy curve *W*(*R*_c_) exhibits a single well with a minimum at *R*_c_ ∼ 5.7 Å, indicative of *open* or *partially closed* M20 loop conformations (Figure 5, dashed curve). As *R*_c_ increases beyond 5.7 Å, the free energy rises sharply, indicating that fully *closed* conformers with *R*_c_ > 5.7 Å are disfavored in such low *I*^M^ solutions. In contrast, as *R*_c_ decreases from 5.7 to −2 Å, the free energy slowly rises by only 1 kcal/mol with apparent dips around *R*_c_ = 1 and −2 Å, corresponding to *occluded* states. However, thermal fluctuations may overcome the tiny barriers separating the *occluded* and *open* states. When *R*_c_ drops below −2 Å, the free energy sharply increases, indicating that conformations with *R*_c_ much less than −2 Å are disfavored. Thus, at low *I*^M^ (0.075 M), the M20 loop in the apoenzyme can explore a relatively wide conformational space spanning *R*_c_ from −2 to 5.7 Å, but it prefers to adopt an *open* or *partially closed* conformation.

#### *I*^M^ = 0.15 M

With an increase in *I*^M^ from 0.075 to 0.15 M, the conformational free energy profiles exhibit oscillations (Figure 5, solid curve). Comparison of the free energy profiles at *I*^M^ = 0.075 and 0.15 M shows that the global minimum, corresponding to *open* M20 loop conformations at *R*_c_ = 5.7 Å, remains unchanged. However, the apparent dip at −1.7 Å seen at *I*^M^ = 0.075 M has developed into a well, representing the *occluded* state. Additionally, another well appeared at *R*_c_ ∼ 2.5 Å. As the barriers separating these minima are less than 1 kcal/mol, thermal fluctuations can lead to the *open* conformer, as for *I*^M^ = 0.075 M. These findings align with the M20 loop conformations seen in *ec*DHFR structures solved at *I*^M^ < 0.25 M, which fall within the global minimum. Interestingly, the *occluded* structure (PDB 1rc4)^19^ solved using *I*^M^ = 0.15 M falls within the well at *R*_c_ ∼ −2 Å.

#### *I*^M^ > 0.25 M

**Figure 6:**
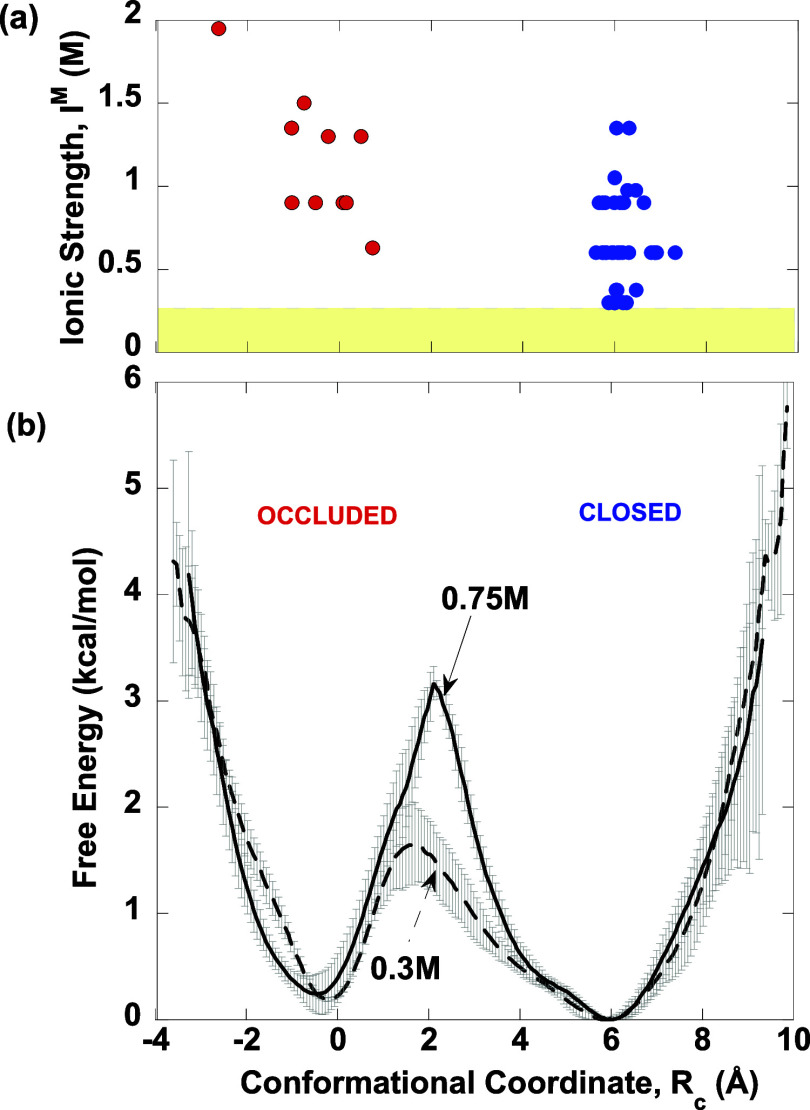
Correlation of free energy profiles for high *I*^M^ (0.30 and 0.75 M) with the *ec*DHFR structures solved at nonphysiological *I*^M^. (a) *Occluded* (*red*) and *closed* (blue) M20 loop conformations classified using hierarchical clustering^20^ in *ec*DHFR PDB structures solved at *I*^M^ > 0.25 M from Figure 4a. (b) Free energy as a function of *R*_c_ at *I*^M^ = 0.3 M (dashed curve) and 0.75 M (solid curve) in CaCl_2_ solution. The gray bars represent the error bars.

At *I*^M^ exceeding the *E. coli* threshold, the computed free energy profiles show two well-defined minima at *R*_c_ of 6 and −0.17 Å corresponding to the *closed* and *occluded* loop states, respectively (Figure 6). As *I*^M^ increased from 0.3 to 0.75 M, the barrier separating the two minima also increased from 1.6 to 3.2 kcal/mol, making transitions between these states less probable. This implies that higher ionic strength enhances the stability of the M20 loop in either the *closed* or *occluded* conformation. Consequently, these states become more stable and can be crystallized at *I*^M^ of 0.3 M or higher. This is in accord with the M20 loop conformations seen in *ec*DHFR structures solved at *I*^M^ > 0.25 M, which predominantly fall into these two free energy wells.

### Enzyme Structural Response to Increasing Ionic Strength

To see how the enzyme responds to increasing ionic strength, we examined the structures at the end of the simulations starting from representative *occluded*, *open*, and *closed* X-ray structures at *I*^M^ = 0.075 and 0.75 M.

#### Starting Occluded Structure (PDB 6cw7)

**Figure 7:**
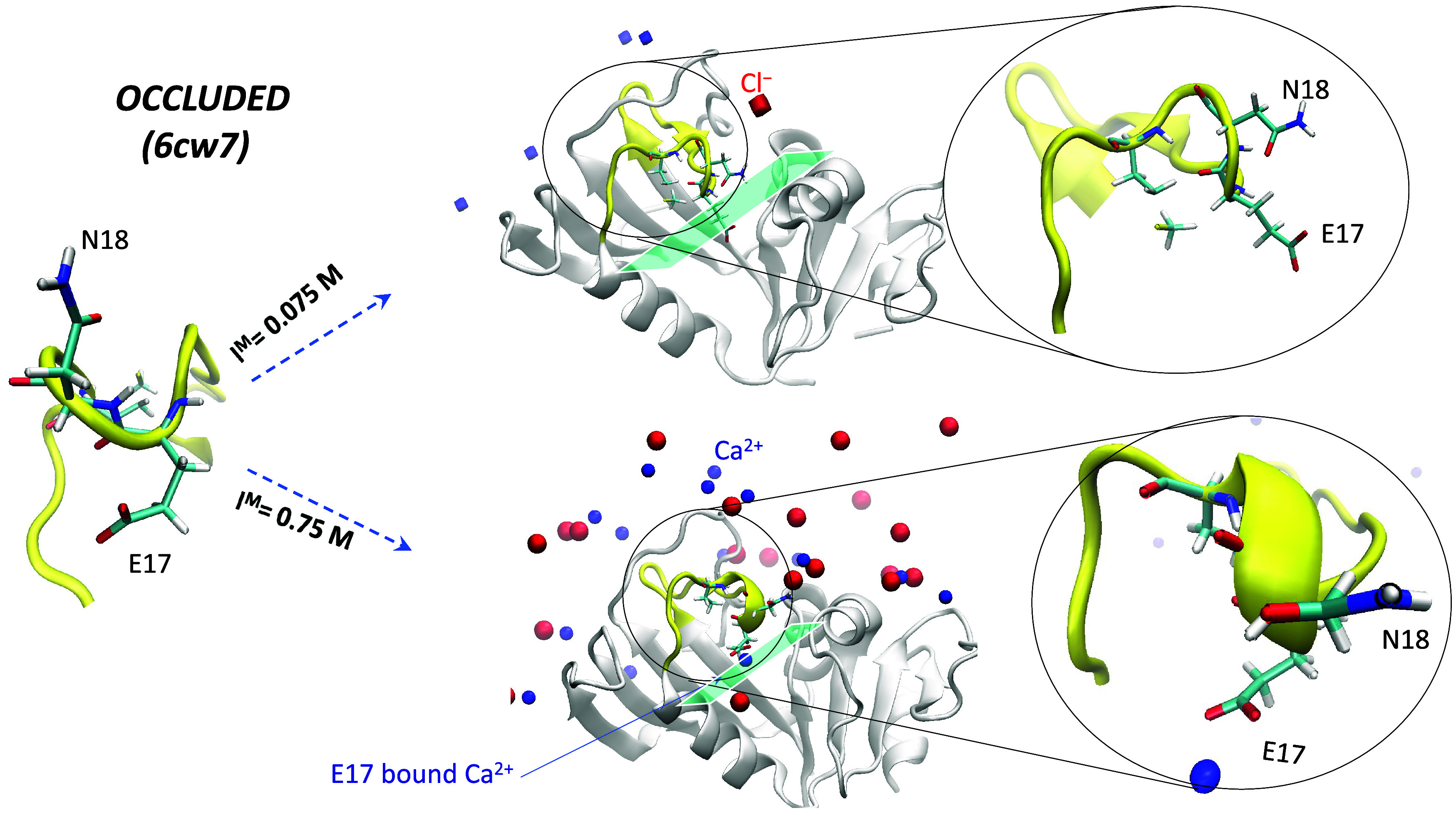
Structures at the end of the simulations starting from representative *occluded* (6cw7) structure at *I*^M^ = 0.075 and 0.75 M. The M20 loop is in yellow with the E17 and N18 loop residues in stick format, whereas Ca^2+^ and Cl^–^ ions are represented by blue and red spheres, respectively. Figures drawn using VMD.^52^

At low *I*^M^, the starting *occluded* M20 loop exhibited unfolding of the 3_10_-helix comprising E17–M20 and flipping of the E17 side chain away from the substrate-binding site into a more *open* structure (Figure 7). This is consistent with the noncanonical *occluded* M20 loop conformation seen in the 1rg7 structure crystallized at low *I*^M^,^19^ where the *R*_c_ is closer to the *R*_c_ range of *open* structures than that of *occluded* structures (Figure 4). In contrast, at higher *I*^M^, the 3_10_-helix was maintained throughout the simulation with the E17 side chain pointing toward the substrate-binding site. Additionally, the E17 carboxylate coordinated Ca^2+^, imparting stability to the *occluded* M20 loop.

#### Starting Open Structure (PDB 1ra2)

**Figure 8:**
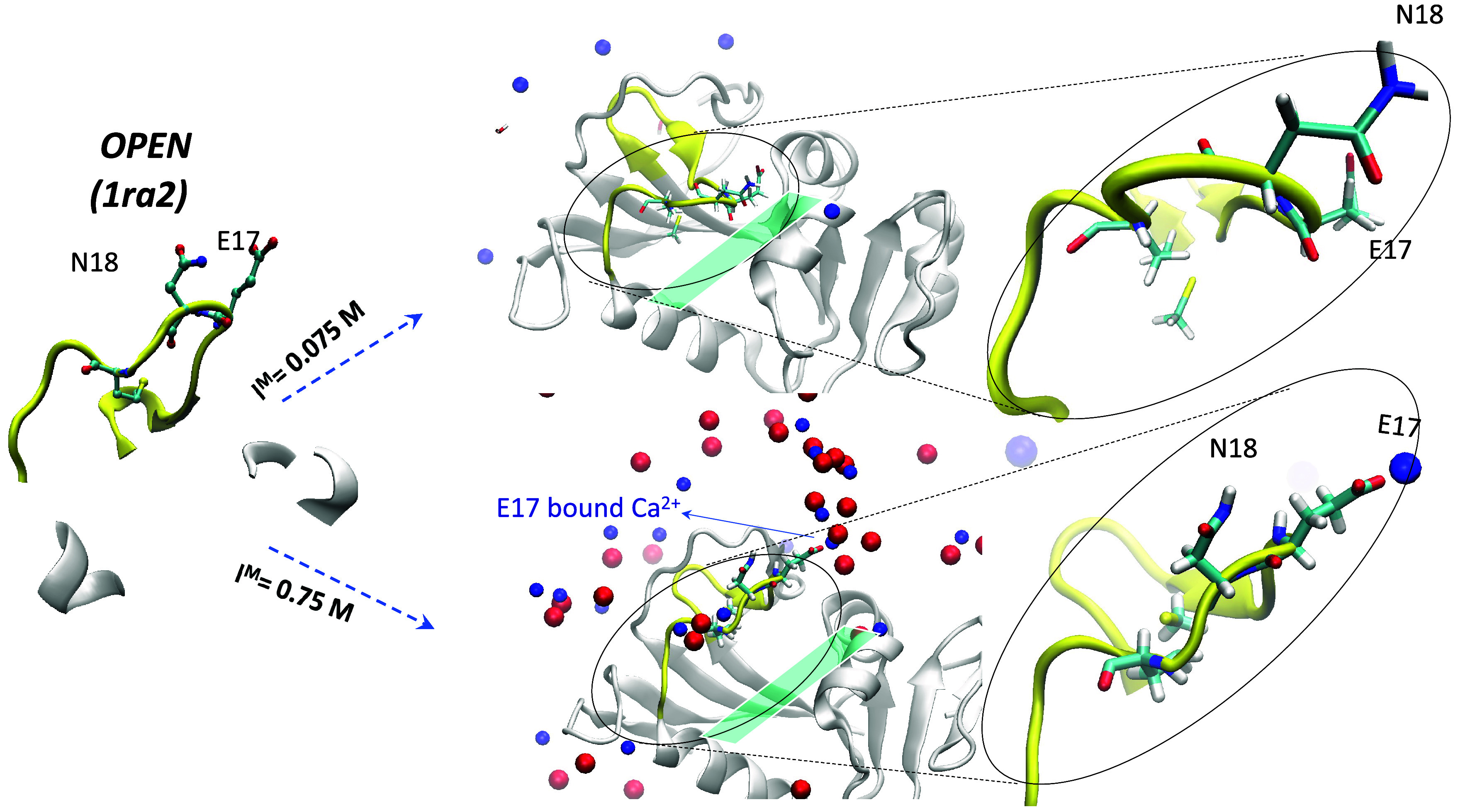
Structures at the end of the simulations starting from representative *open* (1ra2) structure at *I*^M^ = 0.075 and 0.75 M. The M20 loop is in yellow with the E17 and N18 loop residues in stick format, whereas Ca^2+^ and Cl^–^ ions are represented by blue and red spheres, respectively.

Whereas the M20 loop underwent a transition to a more open structure during the simulation starting from the *occluded* structure at low *I*^M^, it did not undergo a transition to the *occluded* form during the simulation starting from the *open*1ra2 structure: The E17 and N18 side chains, despite reorienting, did not flip toward the substrate-binding site or blocked access to the active site (Figure 8). Furthermore, the C^α^–C^α^ distance between N18 and H45 is still longer than that in the *closed* loop structures. At high *I*^M^, the *open* loop conformation persisted throughout the simulation, with both cations and anions surrounding the solvent-exposed loop.

#### Starting Closed Structure (PDB 3ql3)

**Figure 9:**
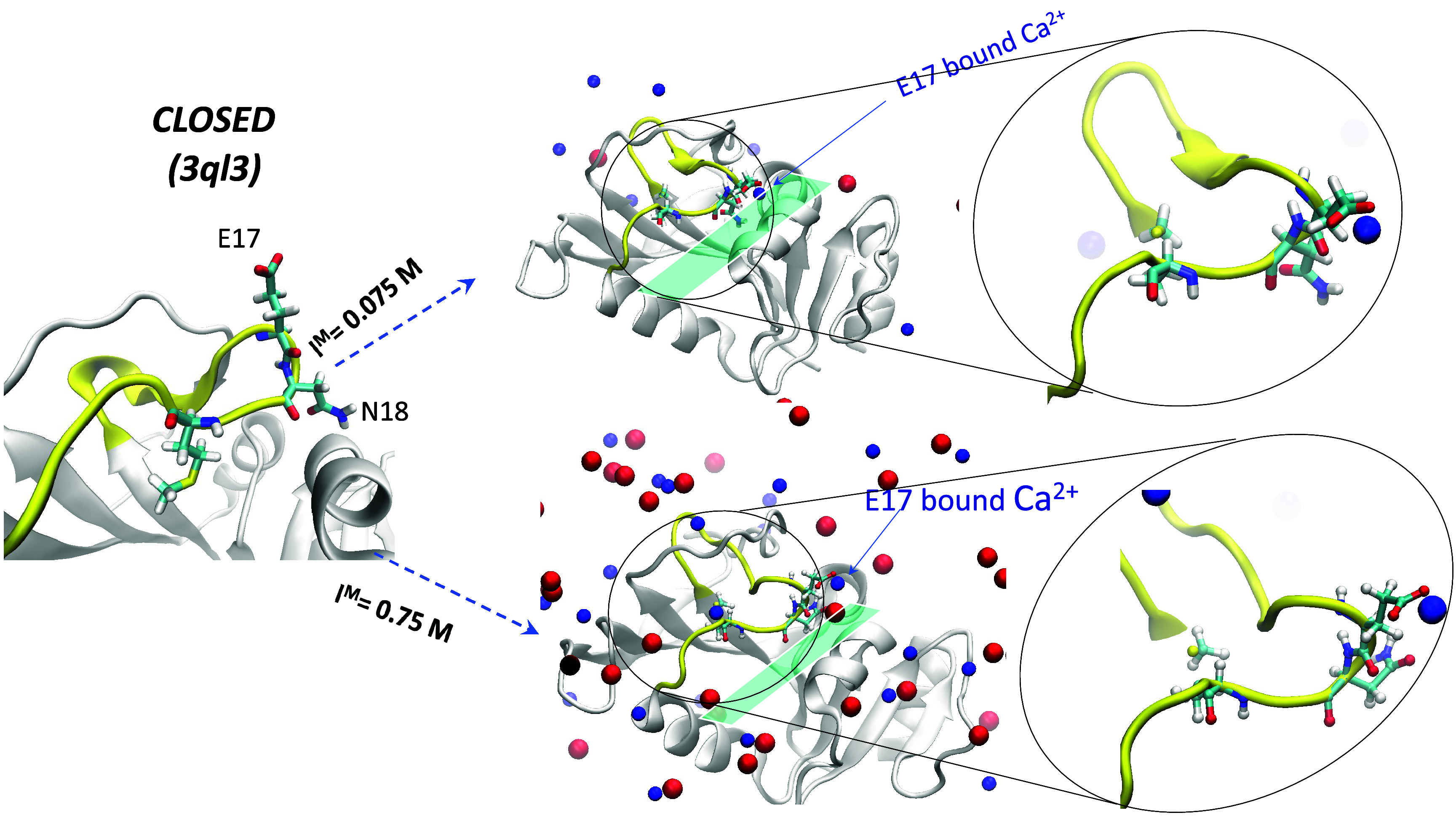
Structures at the end of the simulations starting from representative *closed* (3ql3) structure at *I*^M^ = 0.075 and 0.75 M. The M20 loop is in yellow with the E17 and N18 loop residues in stick format, whereas Ca^2+^ and Cl^–^ ions are represented by blue and red spheres, respectively.

Simulations starting from the *closed* structure preserved the *closed* conformation at both low and high *I*^M^ values (Figure 9). This is likely because the *closed* loop strongly interacts with protein residues around the coenzyme-binding site, so the protein environment can stabilize the *closed* conformation. Furthermore, at high *I*^M^, Ca^2+^ is directly bound to E17, inducing additional stability to the *closed* loop.

### Molecular Basis for M20 Loop Free Energy Modulation by the Ionic Strength

To elucidate the modulation of the M20 loop conformations by the ionic strength, we analyzed the ionic and solvent (water) distributions around a reference point on the loop. This reference point was set at the geometric center of E17, N18, and M20 C^α^ atoms, so it has a net charge of −1e. We computed the ensemble average of distance-dependent electrostatic potential due to ions, Φ^α^(*r*)^ion^, and solvent molecules, Φ^α^(*r*)^solv^, around this reference point on the loop from the simulation trajectories (see the Methods section). From the linear free energy relationship,^53^ half of the total electrostatic potential due to ions, solvent molecules, and protein atoms at a sufficiently large *r*, multiplied by the charge at the reference point, *q*^α^, is related the free energy of the loop, i.e.,
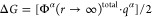
5

Below, we compared ionic and solvent distance-dependent electrostatic potentials at low and high *I*^M^ to obtain insights into the ionic and solvation interactions stabilizing the different loop conformers.

### *I*^M^ = 0.075 M

**Figure 10:**
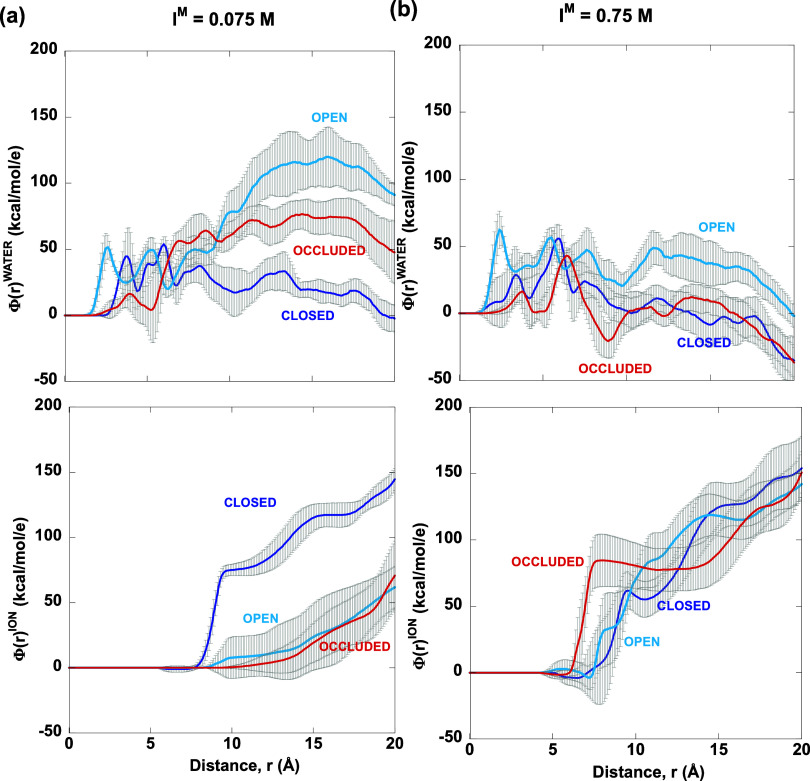
Distance-dependent electrostatic potential due to ions, Φ^α^(*R*)^ion^ and solvent molecules, Φ^α^(*R*)^solv^ as a function of the distance “*r*” from a point representing the geometric average of C_a_ atoms of E17, N18, and ^M^20. (a) *I*^M^ = 0.075 M and (b) *I*^M^ = 0.75 M. For each curve, the gray bars represent the error bars.

The free energy minimum at low *I*^M^ corresponds to an *open* or *partially closed* loop. Compared to the *occluded* or *closed* loop, the *open* loop is better solvated, as evidenced by a closer Φ^α^(*R*)^solv^ first peak at 3 Å and more positive Φ^α^(*R*)^solv^ within a sphere of 20 Å from the reference point (Figure 10a). Apart from water molecules, Ca^2+^ cations may also help to stabilize the *open* M20 loop, which exhibits cationic interactions starting at ∼10 Å. Compared to the *open* loop, the *occluded* loop exhibits a less positive Φ^α^(*r* = 20)^solv^ and the Φ^α^(*r*)^ion^ potential rises only at 14 Å, indicating that ions cannot approach and stabilize the *occluded* loop within this radius. The *closed* M20 loop exhibits cationic interactions starting at 7.5 Å, with dips indicating Cl^–^ interactions, yielding a more positive Φ^α^(*r* = 20)^ion^ than the *occluded* or *open* loop, but the Φ^α^(*r* = 20)^solv^ is ∼0.

### *I*^M^ = 0.75 M

At high *I*^M^, ionic forces are predominant with larger Φ^α^(*r*)^ion^ values than Φ^α^(*r* = 20)^solv^ (Figure 10b). In contrast to low *I*^M^, the *occluded* loop displays strong interactions with Ca^2+^ within an 8 Å radius, with a flat region extending to 14 Å, indicating an absence of ions (Ca^2+^ or Cl^–^). This allows unhindered Ca^2+^ interaction with the loop, distinguishing it from the other conformers, which exhibit oscillations in Φ^α^(*R*)^ion^, indicating interactions with both cations and anions. The *closed* loop exhibits a peak at 9.5 Å due to interactions with Ca^2+^, followed by a dip indicating interactions with Cl^–^. Apart from ionic interactions, solvation interactions also stabilize both *closed* and *occluded* loops, which display similar Φ^α^(*r* = 20)^solv^. In contrast, the *open* loop lacks stabilization by solvation Φ^α^(*r* = 20)^solv^ ∼ 0 and the presence of Cl^–^ ions at a distance of 7 Å may result in unfavorable charge–charge interactions. These differences in ionic and solvent interactions contribute to the emergence of a high free energy barrier between *closed* and *occluded* conformers, which are stabilized by cations and water molecules.

## Discussion and Conclusions

### Ionic Strength-Driven Conformational Transitions

By correlating computed free energy profiles and observed M20 loop conformations in *ec*DHFR PDB structures solved at varying ionic strengths, we reveal the influence of charged species in solution, such as multivalent salts used in crystallization, on the M20 loop conformations. These multivalent ions can interact with the M20 loop directly or collectively and stabilize it in a specific conformation. These solution effects create a substantial free energy barrier (3.2 kcal/mol) between the *closed* and *occluded* states (Figure 6), imparting stability to both conformers. This stabilization would aid in the crystallization of *ec*DHFR with the M20 loop in the *closed* or *occluded* conformation. High ionic strength may promote the formation of a helical/turn structure involving residues 16 to 20, leading to the E17 side chain pointing toward the substrate cavity, as seen in *occluded* structures (Figure 3). In contrast, a lower ionic strength destabilizes this helix, promoting an *open* or partially *closed* loop (Figure 7), consistent with free energy profiles in Figure 5. Altogether, our findings suggest that the observed loop conformations are influenced not only by ligands bound to the enzyme but also by the solution environment.

### Implications for the Catalytic Cycle and Drug Design

Although an *I*^M^ of 0.24 M may not precisely represent the threshold for *E. coli* cells, organisms have an upper limit for *I*^M^, as the concentrations of ions are generally confined to a certain range. At physiological ionic strengths (below 0.24 M), both *open* and *occluded* M20 loop conformations may be accessible, but the *open* conformer is favored in the apoenzyme rather than an *occluded* conformer. Such an *open* loop conformation would enable binding of reactants (NADPH coenzyme and DHF substrate) and unbinding of products (NADPH coenzyme and DHF substrate). On the other hand, it is unclear if an *occluded* conformation, typically seen in structures obtained at *I*^M^ surpassing the *E. coli* threshold, exists *in vivo*. Our findings on the negatively charged M20 loop highlight the need for careful consideration when utilizing X-ray structures, especially of proteins with long functional loops containing charged residues, for structure-based drug design. Such loops may interact with ionic species in solution, resulting in nonphysiological conformations at *I*^M^ surpassing the human threshold. Using such nonphysiological conformations in docking studies may yield inhibitors that could face challenges in clinical trials.

In summary, our study reveals the intricate interplay between solution ionic strength and M20 loop conformations in *ec*DHFR. This novel perspective has implications for understanding the biological significance of loop dynamics, reinterpreting the catalytic cycle, and guiding structure-based drug design strategies for enzymes with charged-residue-containing functional loops.

## Data Availability

Protein structures were downloaded from the Protein Data Bank (PDB). Additional data required for running CHARMM (input, .CRD, and .RST files), and a Fortran code for computing distance-dependent potentials are available at https://github.com/SatheesanBabu/Charmm_run_files_for_Babu_etal_JPCB2024.

## Supporting Information Available

Comparison of free energy profiles from unbiased and umbrella sampling simulations at *I*^M^ of 0.75 M (Figure S1); listing of PDB entries, resolution, pH, type and concentration of salt, the *I*^M^, and the corresponding *R*_c_ values for the *occluded*, *open*, and *closed* M20 loop conformations (Table S1a–c) (PDF)
