# Supplementary material for: Solution Ionic Strength Can Modulate Functional Loop Conformations in E. coli Dihydrofolate Reductase
Source: J Phys Chem B. 2024 Apr 23;128(17):4111–22. doi: 10.1021/acs.jpcb.4c00677 (PMC11075089; doi:10.1021/acs.jpcb.4c00677)
Supplement: Supplementary file 1 — jp4c00677_si_001.pdf [file jp4c00677_si_001.pdf]

Solution Ionic Strength Can Modulate Functional Loop Conformations in *E. coli* Dihydrofolate Reductase

C. Satheesan Babu[†]*, Jih-Ying Chen[†], and Carmay Lim[†,*]

[†]Institute of Biomedical Sciences, Academia Sinica, Taipei 11529, Taiwan

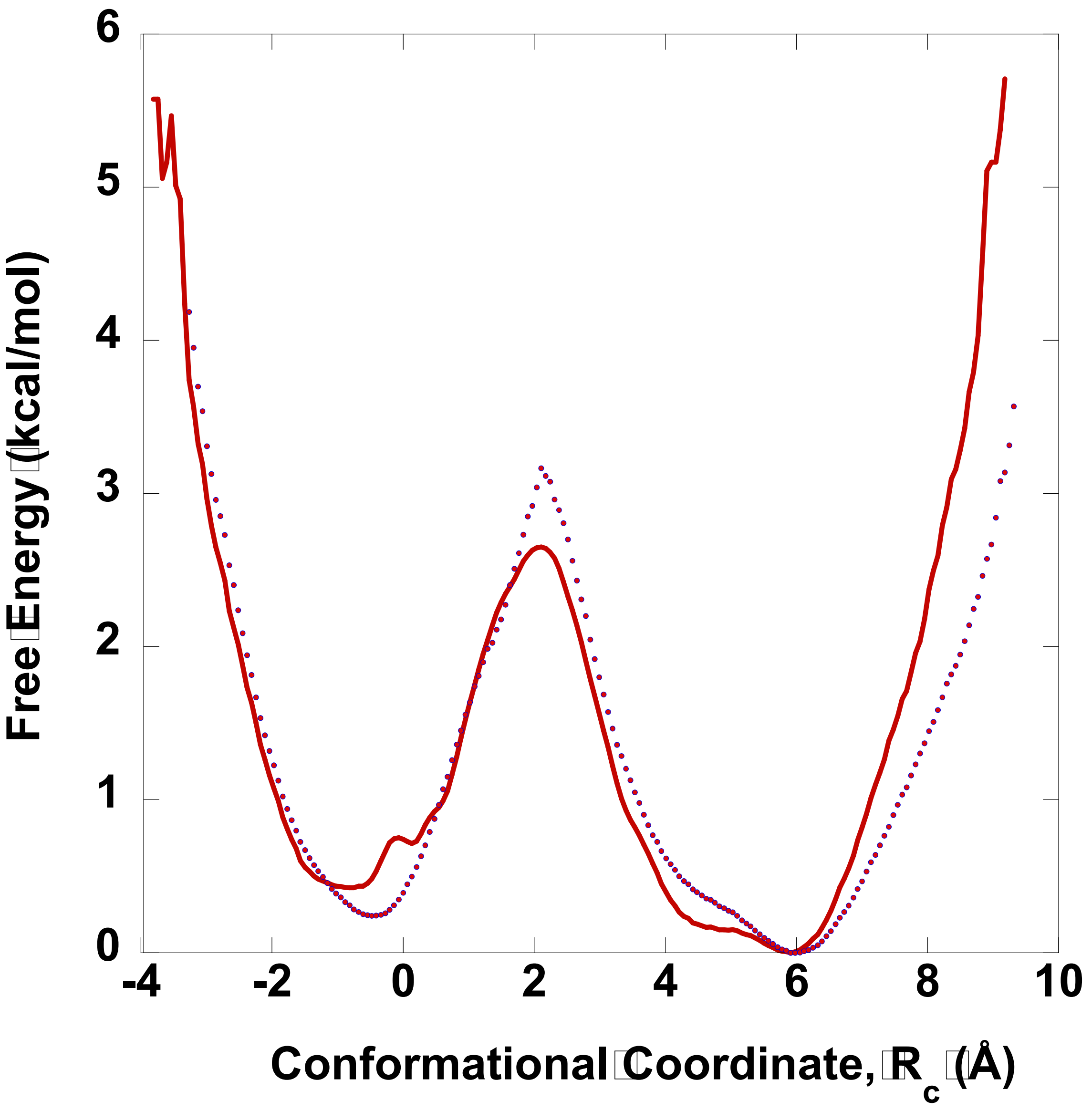

Figure S1. Comparison between free energy profiles from unbiased simulations (dotted curve) and simulations employing umbrella sampling between the two minima at I^M of 0.75M (solid curve).

Table S1a. The PDB entries, resolution, pH, type and concentration of salt, the I^{M} computed using eq 1, and the corresponding R_c values computed using eq 2 for the *occluded* M20 loop.

PDB id	Resolution (Å)	pH	Salt	[Salt] (M)	Ionic Strength (M)	R_c (Å)
1jom	1.9	–	$CaAc_2$	0.5	1.5	–0.77
1rc4	1.9	7.2	$CaCl_2$	0.05	0.15	–0.33
1rf7	1.8	6.6	$CaCl_2$	0.21	0.63	0.72
1rx4	2.0	6.0	$CaCl_2$	0.30	0.9	–1.04
1rx5	2.3	7.4	$MnCl_2$	0.30	0.9	0.08
1rx6	2.0	6.0	$CaCl_2$	0.30	0.9	–0.52
1rx7	2.3	6.5	$MnCl_2$	0.30	0.9	0.15
6cw7	1.03	6.5	$MgCl_2$ + NaCl	0.40	1.3	–0.24
6cxk	1.11	6.5	$MgCl_2$ + NaCl	0.40	1.3	0.47

Table S1b. The PDB entries, resolution, pH, type and concentration of salt, the I^M computed using eq 1, and the corresponding R_c values computed using eq 2 for the *open* M20 loop.

PDB id	Resolution (Å)	pH	Salt	[Salt] (M)	Ionic Strength (M)	R_c (Å)
1ddr_B	2.45	7.0	–	–	0	5.23
1dds_B	2.2	–	–	–	0	5.25
1dhi_B	1.9	–	$CaCl_2$	0.05	0.15	5.31
1dhj_B	1.8	–	–	–	0.0	5.45
1dra_B	1.9	–	$CaCl_2$	0.01	0.03	5.35
1drb_B	1.96	–	$CaCl_2$	0.01	0.03	5.26
1dyh_B	1.90	–	$CaCl_2$	0.05	0.15	5.10
1dyi_B	1.90	–	$CaCl_2$	0.05	0.15	5.03
1dyj_B	1.85	–	$CaCl_2$	0.05	0.15	5.31
1jol_B	1.96	–	–	–	0.0	5.43
1ra1	1.9	7.0	$CaCl_2$	0.011	0.033	4.88
1ra2	1.6	7.0	$CaCl_2$	0.01	0.03	4.95
1ra3	1.8	7.0	$CaCl_2$	0.011	0.033	5.38
1ra8	1.8	7.0	$CaCl_2$	0.01	0.03	4.83
1ra9	1.55	7.0	$CaCl_2$	0.011	0.033	4.99
1rb2_A	2.1	8.0	$CaCl_2$	0.09	0.27	4.92
1rb2_B	2.1	8.0	$CaCl_2$	0.09	0.27	4.99
1re7_A	2.6	8.0	$MgCl_2$	0.2	0.60	5.07
1re7_B	2.6	8.0	$MgCl_2$	0.2	0.60	4.94
1rg7	2.0	7.2	$CaCl_2$	0.05	0.15	4.16
1tdr_B	2.5	–	$CaCl_2$	0.05	0.0	5.55
2drc_B	1.9	6.8	$CaAc_2$	0.05	0.15	5.30
3drc_B	1.9	–	$CaCl_2$	0.005	0.15	5.54
4x5f_A	1.7	–	$CaCl_2$	0.01	0.03	5.21
4x5f_B	1.7	–	$CaCl_2$	0.01	0.03	5.15
4x5g_A	1.9	–	$CaCl_2$	0.01	0.03	5.19
4x5g_B	1.9	–	$CaCl_2$	0.01	0.03	5.12
4x5h	1.9	–	$CaCl_2$	0.01	0.03	4.64
4x5i	1.8	–	$CaCl_2$	0.01	0.03	4.76
4x5j	1.85	–	$CaCl_2$	0.01	0.03	4.73
5uip_A	1.9	–	–	–	0.0	5.31
5uip_B	1.9	–	–	–	0.0	5.52

Table S1c. The PDB entries, resolution, pH, type and concentration of salt, the I^M computed using eq 1, and the corresponding R_c values computed using eq 2 for the *closed* M20 loop.

PDB id	Resolution (Å)	pH	Salt	[Salt] (M)	Ionic Strength (M)	R_c (Å)
1ddr_A	2.45	7.0	–	–	0.0	7.12
1dds_A	2.2	–	–	–	0.0	6.90
1dhi_A	1.9	–	$CaCl_2$	0.05	0.15	6.94
1dhj_A	1.8	–	–	–	0.0	6.92
1dra_A	1.9	–	$CaCL_2$	0.01	0.03	6.85
1drb_A	1.96	–	$CaCL_2$	0.01	0.03	6.78
1dre	2.0	6.0	$CaCL_2$	0.01	0.03	6.18
1drh	2.3	7.0	$CaCl_2$	0.008	0.024	6.38
1dyh_A	1.9	–	$CaCl_2$	0.05	0.15	6.71
1dyi_A	1.85	–	$CaCl_2$	0.05	0.15	6.65
1dyj_A	1.85	–	$CaCl_2$	0.05	0.15	6.72
1jol_A	1.96	–	–	–	0.0	6.92
1rb3_A	2.3	7.0	–	–	0.0	5.68
1rb3_B	2.3	7.0	–	–	0.0	6.14
1rh3	2.4	7.0	$CaCl_2$	0.008	0.024	6.27
1rx1	2.0	8.0	$CaCl_2$	0.45	1.35	6.04
1rx2		7.0	$MnCl_2$	0.30	0.90	6.20
1rx3		7.0	$MnCl_2$	0.30	0.90	6.13
1rx8	2.8	7.0	$MnCl_2$	0.30	0.90	6.00
1rx9	–	7.0	$CaCl_2$	0.30	0.9	5.79
1tdr_A	2.5	–	$CaCl_2$	0.05	0.15	6.94
2ano	2.68	7.5	$CaCl_2$	0.30	0.9	5.66
2anq	2.13	7.5	$CaCl_2$	0.30	0.9	5.74
2drc_A	1.9	6.8	$CaCl_2$	0.05	0.15	6.94
2inq_A	2.2	7.5	$CaCl_2$	0.02	0.6	7.32
2inq_B	2.2	7.5	$CaCl_2$	0.02	0.6	5.59
3drc_A	1.9	–	$CaCl_2$	0.05	0.015	6.93
3kfy	2.08	8.0	$CaCl_2$	0.35	1.05	6.00
3ql0	1.6	7.5	$CaCl_2$	0.2	0.6	6.80
3ql3	1.8	7.5	$CaCl_2$	0.2	0.6	6.31
3qyl	1.79	8.0	$CaCl_2$	0.325	0.975	6.46
3qyo	2.09	8.0	$CaCl_2$	0.325	0.975	6.28
3r33	2.09	8.0	$CaCl_2$	0.30	0.9	6.64
4dfr_A	–	–	–	–	0.0	6.93
4dfr_B	–	–	–	–	0.0	5.66
4kjj	1.15	7.5	$MgCl_2$	0.20	0.60	5.75
4kjk	1.35	7.5	$MgCl_2$	0.20	0.60	5.75

4kjl	1.38	7.5	$MgCl_2$	0.20	0.60	5.97
4nx6	1.35	7.5	$MgCl_2$	0.20	0.60	6.17
4nx7	1.15	7.5	$MgCl_2$	0.20	0.60	6.11
4p66	1.84	6.5	$CaAc_2$	0.10	0.30	6.00
4p68	2.26	6.5	CaAc2	0.05	0.15	6.54
4pdj	1.6	7.0	$MnCl_2$	0.10	0.30	6.18
4pss	0.85	7.0	$MnCl_2$	0.125	0.375	6.03
4pst	1.05	7.0	$MnCl_2$	0.125	0.375	6.47
4psy	0.85	7.0	$MnCl_2$	0.125	0.375	6.05
4rgc		7.0	$MnCl_2$	0.125	0.375	6.03
5ujx	1.8	7.5	$CaCl_2$	0.20	0.6	6.87
5z6f	1.8	–	$CaCl_2$	0.10	0.3	6.26
5z6j	1.8	–	$CaCl_2$	0.10	0.3	5.99
5z6l	1.9	–	$CaCl_2$	0.10	0.3	6.01
5z6k	1.8	–	$CaCl_2$	0.10	0.3	5.87
5w3q	1.4	8.0	$CaCl_2$	0.45	1.35	6.31
7dfr	2.3	–	$CaCl_2$	0.011	0.033	6.02
